# Metformin induces a fasting- and antifolate-mimicking modification of systemic host metabolism in breast cancer patients

**DOI:** 10.18632/aging.101960

**Published:** 2019-05-09

**Authors:** Elisabet Cuyàs, Salvador Fernández-Arroyo, Maria Buxó, Sonia Pernas, Joan Dorca, Isabel Álvarez, Susana Martínez, Jose Manuel Pérez-Garcia, Norberto Batista-López, César A. Rodríguez-Sánchez, Kepa Amillano, Severina Domínguez, Maria Luque, Idoia Morilla, Agostina Stradella, Gemma Viñas, Javier Cortés, Sara Verdura, Joan Brunet, Eugeni López-Bonet, Margarita Garcia, Samiha Saidani, Jorge Joven, Begoña Martin-Castillo, Javier A. Menendez

**Affiliations:** 1 Program Against Cancer Therapeutic Resistance (ProCURE), Metabolism and Cancer Group, Catalan Institute of Oncology, Girona, Spain; 2 Girona Biomedical Research Institute (IDIBGI), Girona, Spain; 3 Unitat de Recerca Biomèdica, Hospital Universitari de Sant Joan, IISPV, Rovira i Virgili University, Reus, Spain; 4 Department of Medical Oncology, Breast Unit, Catalan Institute of Oncology-Hospital Universitari de Bellvitge-Bellvitge Research Institute (IDIBELL), L’Hospitalet de Llobregat, Barcelona, Spain; 5 Medical Oncology, Catalan Institute of Oncology, Girona, Spain; 6 Medical Oncology Service, Hospital Universitario Donostia, Donostia-San Sebastián, Spain; 7 Biodonostia Health Research Institute, Donostia-San Sebastián, Spain; 8 Medical Oncology Department, Hospital de Mataró, Mataró, Barcelona, Spain; 9 IOB Institute of Oncology, Hospital Quirónsalud, Madrid and Barcelona, Spain; 10 Medical Oncology Service, Hospital Universitario de Canarias, La Laguna, Tenerife, Spain; 11 Medical Oncology Service, Hospital Universitario de Salamanca, Salamanca, Spain; 12 Instituto de Investigación Biomédica de Salamanca (IBSAL), Salamanca, Spain; 13 Medical Oncology, Hospital Universitari Sant Joan, Reus, Spain; 14 Medical Oncology Service, Hospital Universitario Araba, Vitoria-Gasteiz, Spain; 15 Department of Medical Oncology, Hospital Universitario Central de Asturias, Oviedo, Spain; 16 Vall d’Hebron Institute of Oncology (VHIO), Barcelona, Spain; 17 Hereditary Cancer Programme, Catalan Institute of Oncology (ICO), Bellvitge Institute for Biomedical Research (IDIBELL), L’Hospitalet del Llobregat, Barcelona, Spain; 18 Hereditary Cancer Programme, Catalan Institute of Oncology (ICO), Girona Biomedical Research Institute (IDIBGI), Girona, Spain; 19 Department of Anatomical Pathology, Dr. Josep Trueta Hospital of Girona, Girona, Spain; 20 Clinical Research Unit, Catalan Institute of Oncology, L’Hospitalet de Llobregat, Barcelona, Spain; 21 Unit of Clinical Research, Catalan Institute of Oncology, Girona, Spain; * Equal contribution

**Keywords:** metformin, breast cancer, ketogenic diet, ketone bodies, homocysteine

## Abstract

Certain dietary interventions might improve the therapeutic index of cancer treatments. An alternative to the “drug plus diet” approach is the pharmacological reproduction of the metabolic traits of such diets. Here we explored the impact of adding metformin to an established therapeutic regimen on the systemic host metabolism of cancer patients. A panel of 11 serum metabolites including markers of mitochondrial function and intermediates/products of folate-dependent one-carbon metabolism were measured in paired baseline and post-treatment sera obtained from HER2-positive breast cancer patients randomized to receive either metformin combined with neoadjuvant chemotherapy and trastuzumab or an equivalent regimen without metformin. Metabolite profiles revealed a significant increase of the ketone body β-hydroxybutyrate and of the TCA intermediate α-ketoglutarate in the metformin-containing arm. A significant relationship was found between the follow-up levels of homocysteine and the ability of treatment arms to achieve a pathological complete response (pCR). In the metformin-containing arm, patients with significant elevations of homocysteine tended to have a higher probability of pCR. The addition of metformin to an established anti-cancer therapeutic regimen causes a fasting-mimicking modification of systemic host metabolism. Circulating homocysteine could be explored as a clinical pharmacodynamic biomarker linking the antifolate-like activity of metformin and biological tumor response.

## Introduction

Tumor heterogeneity often reduces the efficacy of both non-targeted and genome-driven targeted cancer therapies [1–4]. Deregulated cellular metabolism is a trait shared by virtually all tumor cells across multiple cancer types, and might be exploited to bypass this therapeutic limitation [5–8]. However, given the intrinsic metabolic flexibility of cancer cells, targeting specific metabolic pathways might be just as challenging as targeting somatic mutations, if not more so [9–13]. A higher anti-cancer potential might arise from combining standard treatments with specific dietary interventions which, by changing the levels of certain host metabolites, would restrict the usage of alternative signaling and metabolic nodes by cancer cells [14]. Although originally assumed not to be relevant, the possibility that specific dietary interventions can influence the outcome of some cancer treatments is beginning to be recognized in pre-clinical and clinical scenarios.

Restriction of the amino acids serine and glycine in the diet increases the survival of cancer-prone mice [15], and provides a plausible explanation for the recognized anti-cancer effects of low-protein diets or dietary restriction [16]. Furthermore, the high-fat low-protein/carbohydrate ketogenic diet (KD), which increases blood ketones such as β-hydroxybutyrate (BHBA) and decreases blood glucose by simulating the physiological response to fasting, greatly enhances the efficacy/toxicity ratios of PI3K inhibitors in animal models [17]. Finally, the efficacy of lower, non-toxic doses of the anti-folate methotrexate can be improved through a simple dietary supplementation of histidine [18]. Such experimental confirmation that harnessing dietary metabolic pathways can augment the effects of cancer drugs has received a high degree of social media attention as it highlights that a careful scientific examination of diet as (complementary) medicine is long overdue in oncology. Not surprisingly, combinations of dietary approaches including fasting or low-calorie fasting-mimicking diets (FMD) and KD with chemotherapy, immunotherapy or other cancer treatments are beginning to be viewed as potentially promising strategies to reduce treatment-related adverse effects and boost efficacy outcomes [14]. However, it should be acknowledged that an established indication of FMD or KD, which could decrease protein-calorie intake during oncology treatments, is not yet available and caution has been raised given the prevalence of malnutrition and sarcopenia in patients with cancer [19].

An alternative to this “drug plus diet” approach is the use of pharmacological interventions with low toxicity profiles that can reproduce the metabolic features associated with these diets (e.g., lowering glucose/insulin/IGF1 and increasing ketone bodies). One such pharmacological mimetic is metformin, a biguanide drug commonly used to treat type 2 diabetes and which was originally identified as a putative dietary restriction-mimetic that reproduced the hepatic gene expression profiles shaped by long-term calorie restriction in mice [20,21]. Global metabolomic profiling suggests that metformin might promote a KD-like signature of fatty acid oxidation involving significant increases of BHBA and also of tricarboxylic acid (TCA) cycle intermediates in patients with endometrial cancer [22] and in people with Li-Fraumeni syndrome, who are predisposed to various cancers [23]. Although a few studies have explored the metformin-related metabolic responses in ovarian cancer patients who were receiving metformin for diabetes [24], or in treatment-naïve preoperative window clinical trials in endometrial and breast cancer [22,25], there is no evidence of the impact of adding metformin to established treatment regimens on systemic metabolic markers in everyday oncology practice.

Here we explored the impact of metformin on serum metabolic profiles of patients participating in the METTEN study, a phase 2 clinical trial of HER2-positive breast cancer patients randomized to receive either metformin combined with anthracycline/taxane-based chemotherapy and trastuzumab or an equivalent regimen without metformin, before surgery [26]. A panel of 11 metabolites was selected based on the DR-mimetic [22] and one-carbon (1C) metabolism anti-folate-like activities of metformin [27–30], and included BHBA and the key TCA cycle intermediate α-ketoglutarate, and also intermediates or products of 1C metabolism (i.e., cystathionine, taurine, betaine, choline, dimethylglycine, homocysteine, methionine, s-adenosyl methionine [SAM], and s-adenosyl homocysteine [SAH]).

## RESULTS

### Study participants

To investigate the metabolic changes associated with adding metformin to an anthracycline/taxane-based chemotherapy and trastuzumab regimen, we conducted the present study with paired baseline and post-treatment serum samples collected from 68 patients belonging to the intention-to-treat population of the METTEN trial, which included randomly assigned patients receiving at least one dose of study medication [26]. The baseline characteristics of these patients are shown in Table 1. The comparison of clinical-pathological variables of each cohort revealed no significant differences.

**Table 1:** Baseline patient demographic and tumor characteristics.

		**Metformin arm (*n*=33)**		**Standard arm (*n*=35)**		**p*-value***
**Age (years)**						0.649
	<50	18 (54.5%)		21 (60.0%)		
	≥50	15 (45.5%)		14 (40.0%)		
	Mean ± SD (range)	48.6 ± 10.2 (32–75)		49.1 ± 11.0 (30–72)		0.843
**Menopausal status**						0.772
	Post	13 (39.4%)		15 (42.9%)		
	Pre	20 (60.6%)		20 (57.1%)		
**Body weight (kg)**						
	Mean ± SD (range)	65.8 ± 7.8 (52–89)		65.3 ± 9.6 (48–83)		0.806
**Body mass index**						0.467
	<25	18 (54.5%)		16 (45.7%)		
	≥25 (overweight)	15 (45.5%)		19 (54.4%)		
**Clinical tumor status**						0.750^1^
	cT2	21 (63.6%)		21 (60.0%)		
	cT3	11 (33.3%)		10 (28.6%)		
	cT4b	1 (3.0%)		3 (8.6%)		
	cT4d	0 (0.0%)		1 (2.9%)		
**Clinical nodal stage**						0.414^1^
	cN0	8 (24.2%)		11 (31.4%)		
	cN1	21 (63.6%)		16 (45.7%)		
	cN2	1 (3.0%)		4 (11.4%)		
	cN3	3 (9.1%)		4 (11.4%)		
**Hormone receptor status**						1.000
	ER and/or PgR positive	18 (54.5%)		19 (54.3%)		
	ER and PR negative	15 (45.5%)		16 (45.7%)		
**Tumor grade**						0.467^1^
	G1	2 (7.7%)		0 (0.0%)		
	G2	12 (46.2%)		14 (48.3%)		
	G3	12 (46.2%)		15 (51.7%)		

^1^ Fisher´s exact test

### Addition of metformin elevates circulating levels of β-hydroxybutyrate and α-ketoglutarate in breast cancer patients treated with a conventional neoadjuvant schedule

**Figure 1:**
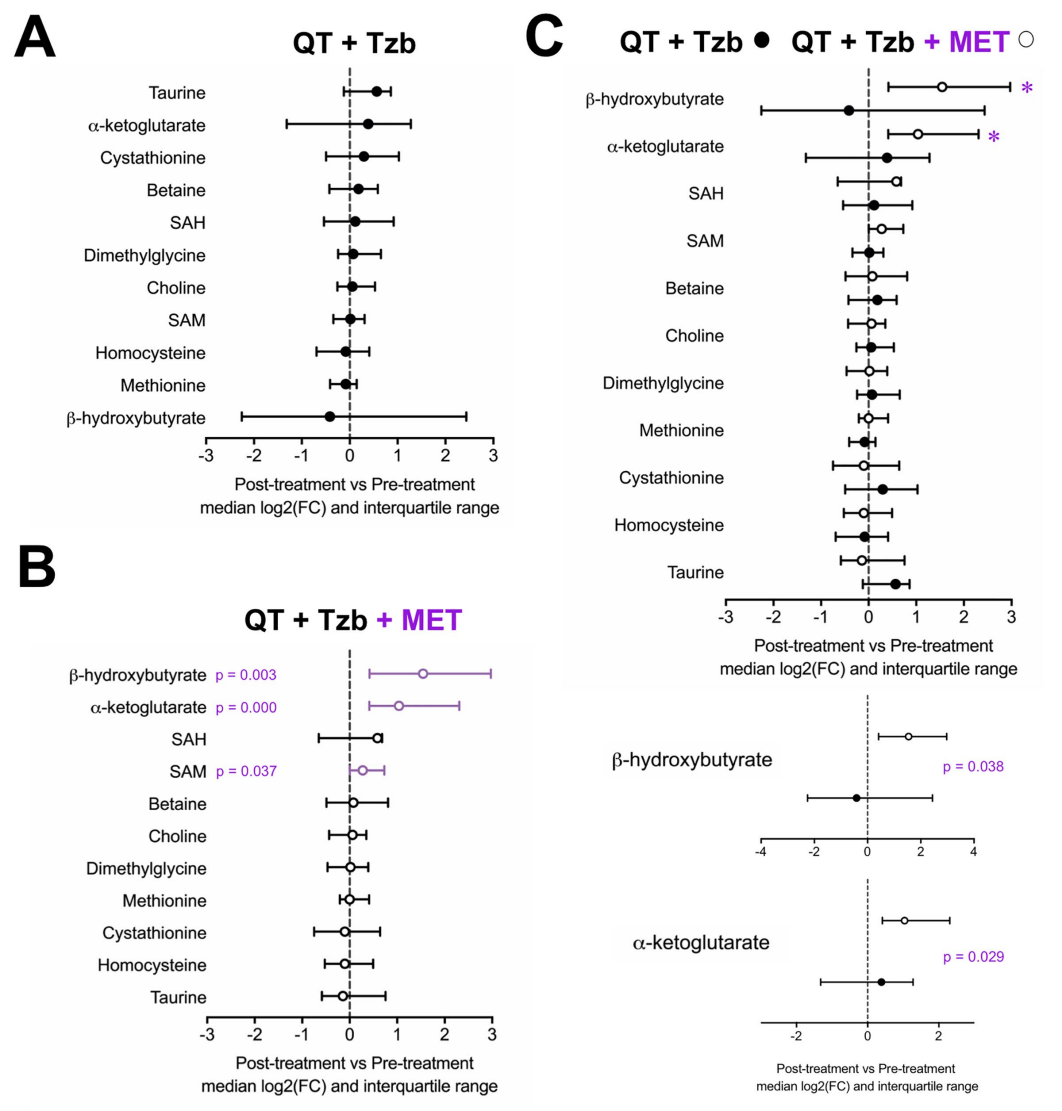
Median fold-change and interquartile range for circulating metabolites (post-treatment *vs* pre-treatment) in the standard neodjuvant regimen arm (**A**), the metformin plus standard regimen arm (**B**), and in patients on metformin compared with those not exposed to metformin (**C**). Metabolites with statistically significant absolute change on Wilcoxon signed rank test are shown with p-values.

To probe the specific metabolic response associated with metformin, the serum profiles of BHBA, α-ketoglutarate, cystathionine, taurine, betaine, choline, dimethylglycine, homocysteine, methionine, SAM, and SAH were first evaluated within each arm of the study. An inspection of the metabolite profile by comparing the median fold-change (post-treatment *vs* pre-treatment) revealed that none of the measured metabolites showed differences in those patients receiving the standard neoadjuvant arm without metformin (Figure 1A). In the metformin-containing arm, however, we observed a statistically significant increase in the serum levels of BHBA (p=0.003), α-ketoglutarate (p=0.000), and SAM (p=0.037) post-treatment (Figure 1B). When we evaluated the differential impact on serum metabolic profiles between treatment arms, only BHBA (p=0.038), and α-ketoglutarate (p=0.029) reached statistical significance (Figure 1C).

### Metformin-driven increase of BHBA is higher in breast cancer patients achieving pathological complete response.

**Figure 2:**
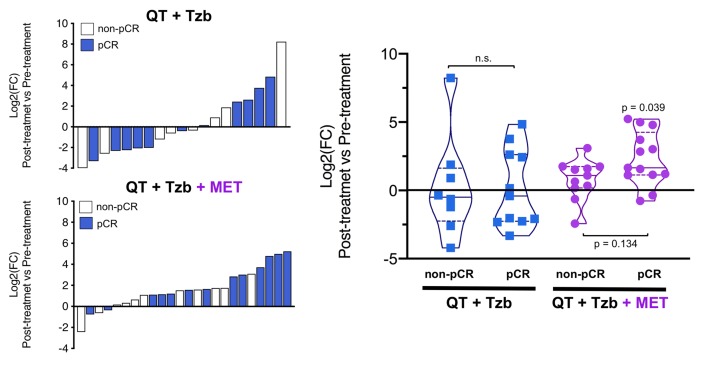
*Left.* Waterfall plots showing the log2 fold chance of circulating BHBA and correlation with treatment outcomes. *Right.* Violin plots depicting the log2 fold chance of circulating BHBA in each treatment arm categorized by treatment outcomes. (pCR: pathological complete response; QT: chemotherapy; Tzb: trastuzumab; MET: metformin; p-values by Wilcoxon signed-ranked test).

**Figure 3:**
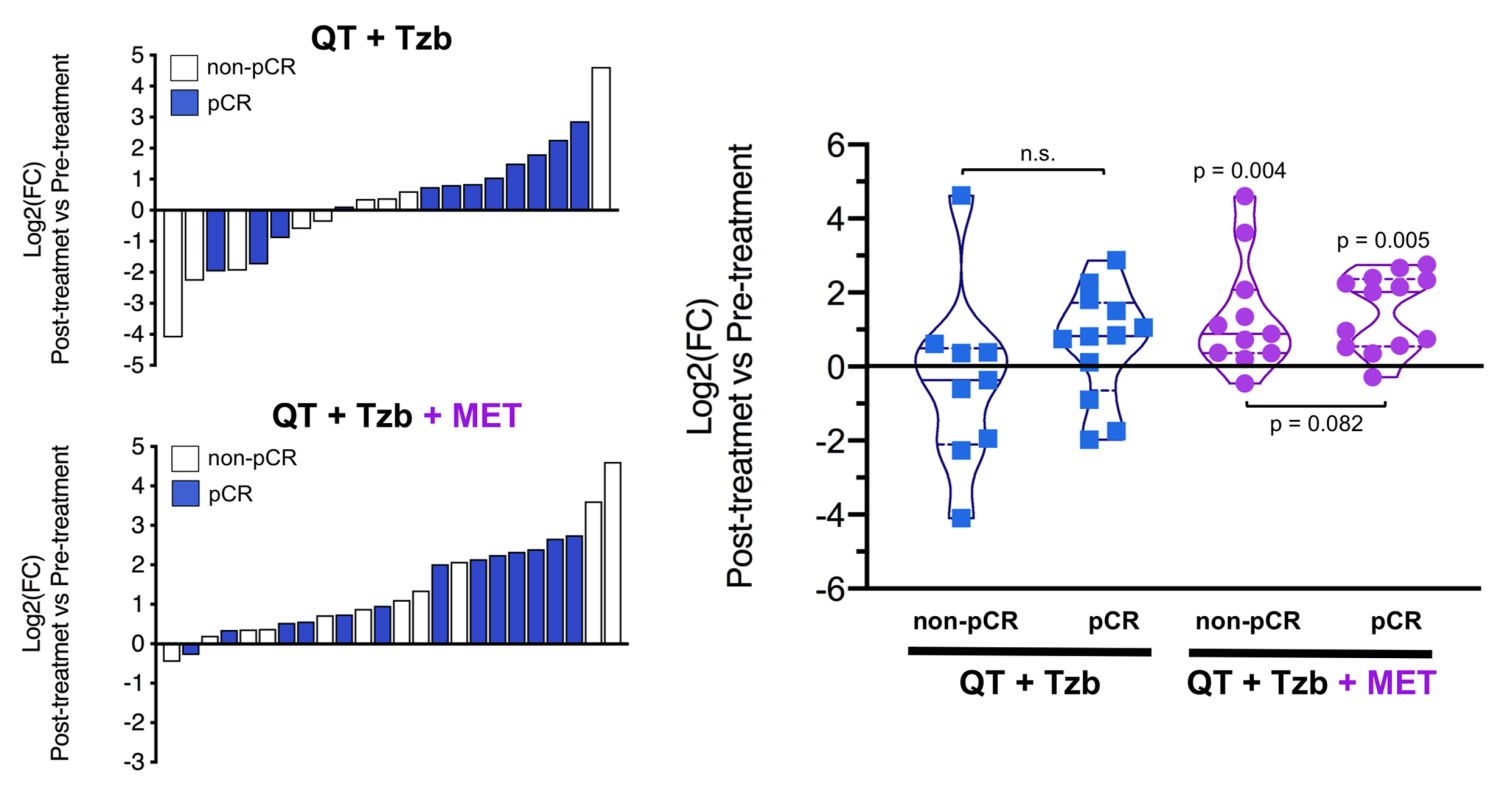
*Left.* Waterfall plots showing the log2 fold chance of circulating α-KG and correlation with treatment outcomes. *Right.* Violin plots depicting the log2 fold chance of circulating α-KG in each treatment arm categorized by treatment outcomes. (pCR: pathological complete response; QT: chemotherapy; Tzb: trastuzumab; MET: metformin; p-values by Wilcoxon signed-ranked test).

**Figure 4:**
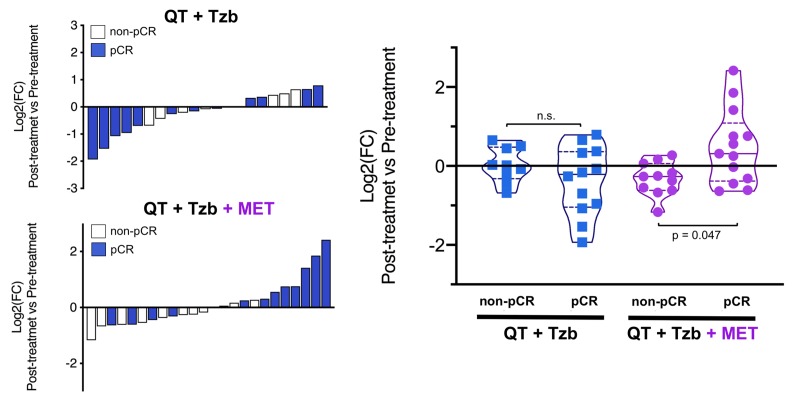
*Left.* Waterfall plots showing the log2 fold chance of circulating Hcy and correlation with treatment outcomes. *Right.* Violin plots depicting the log2 fold chance of circulating Hcy in each treatment arm categorized by treatment outcomes. (pCR: pathological complete response; QT: chemotherapy; Tzb: trastuzumab; MET: metformin; p-values by Wilcoxon signed-ranked test).

The fold-changes in serum levels of BHBA, α-ketoglutarate, and homocysteine in patients achieving or not pCR in the two treatment arms are represented as waterfall and violin plots in Figures 2, 3, and 4 respectively. The fold-change increase of circulating BHBA reached statistical significance in metformin-treated patients achieving pCR, but not in non-pCR patients (Figure 2). By contrast, the fold-change increase of α-ketoglutarate reached statistical significance in metformin-treated patients irrespective of their pCR status (Figure 3). Finally, metformin-treated patients achieving pCR had significantly higher levels of circulating homocysteine than non-pCR patients (p=0.047; Figure 4).

### Follow-up homocysteine predicts the likelihood to benefit from adding pre-operative metformin

**Figure 5:**
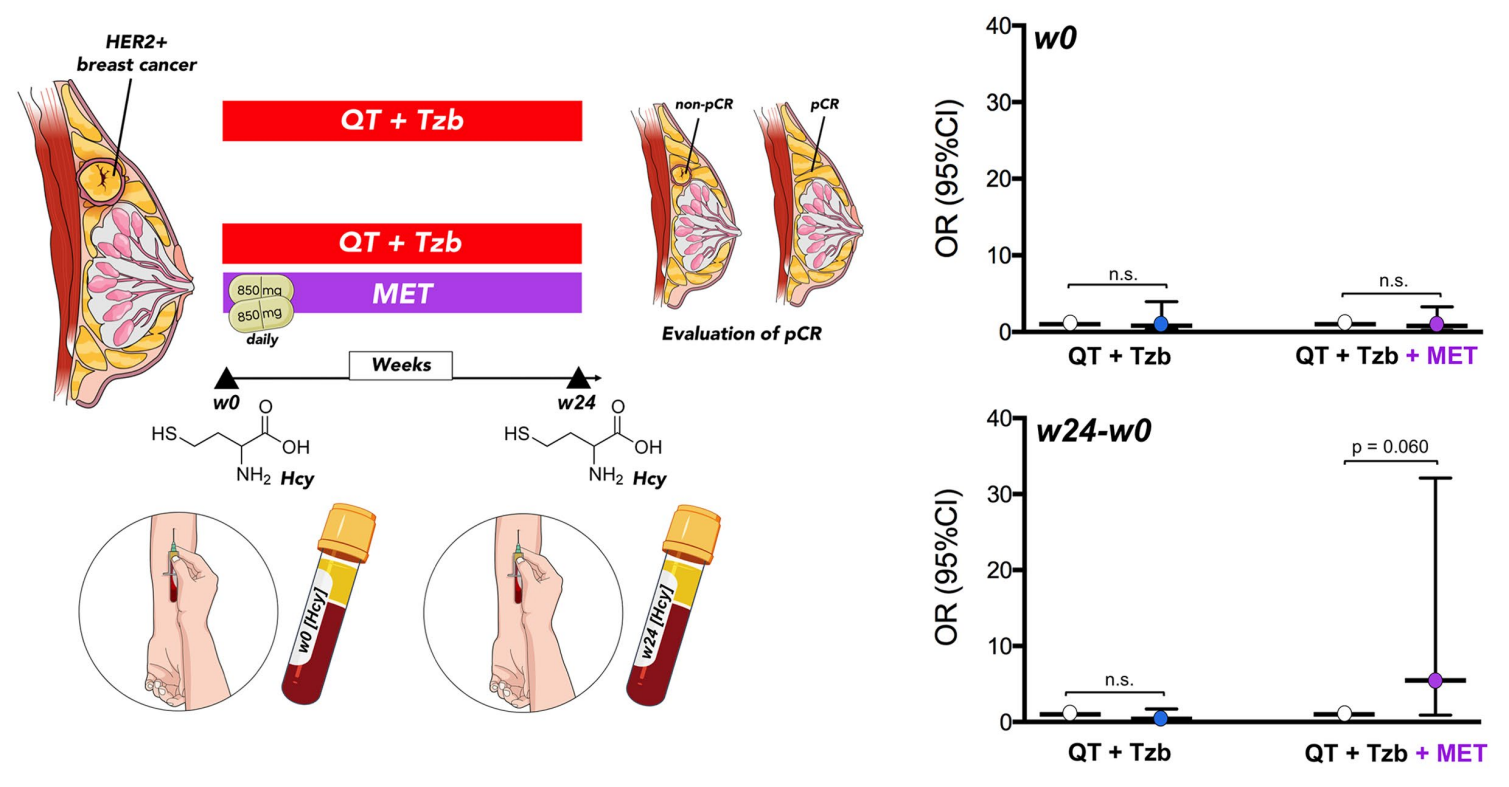
Relationship between the baseline (w0) and the follow-up (w24 minus w0) levels of circulating Hcy and the ability of treatment arms to achieve pCR. (w: week).

Baseline levels of serum homocysteine (week 0 [w0]) were not significantly associated with pCR in patients (Table 2, Figure 5). However, we observed a significant relationship between the follow-up levels of homocysteine (i.e., post-treatment [w24] minus pre-treatment [w0]) and the ability of the treatment arms to achieve pCR (odds ratio [OR]_follow-up homocysteine × arm_ = 13.42, 95% confidence interval [CI]: 1.37–130.98, p=0.025; Table 2). Accordingly, those patients with higher levels of homocysteine in the metformin-containing arm tended to have a higher probability of pCR (OR = 5.47, 95%CI: 0.93–32.11, p=0.060; Figure 5).

**Table 2:** Association of the interaction between baseline and follow-up levels of circulating homocysteine and pathological complete response by treatment arm.

	**Odds ratio (95%CI)**	**p-value**
**Baseline homocysteine (w0)**	1.162 (0.340–3.965)	0.811
Treatment arm	0.658 (0.241–1.792)	0.412
Homocysteine × treatment arm	0.706 (0.110–4.516)	0.713
		
Homocysteine w0 standard arm	1.162 (0.340–3.965)	0.811
Homocysteine w0 metformin arm	0.820 (0.204–3.298)	0.780
		
**Follow-up homocysteine (w24-w0)**	0.408 (0.097–1.714)	0.221
Treatment arm	0.825 (0.226–3.009)	0.771
Homocysteine × treatment arm	**13.419** (1.375–130.982)	**0.025**
		
Homocysteine w24-w0 standard arm	0.408 (0.097–1.714)	0.221
Homocysteine w24-w0 metformin arm	**5.474** (0.933–32.108)	**0.060**

After additional adjustments for potential confounding tumor characteristics, such as tumor size and hormone receptor status, the relationship between the follow-up levels of homocysteine and the ability of treatment arms to achieve a pCR in patients remained significant (adjusted OR_follow-up homocysteine × arm_ = 47.58, 95%CI: 1.60–1411.93, p=0.026; Table 3). In the metformin-containing arm, the positive association between circulating follow-up homocysteine and pCR maintained a tendency towards significance (p=0.076) after accounting for tumor size and hormone receptor status (Table 3). The lack of association between circulating follow-up homocysteine and pCR in the (non-metformin) reference arm remained after adjusting for these factors (Table 3).

**Table 3:** Association of the interaction between baseline and follow-up levels of circulating homocysteine and pathological complete response by treatment arm adjusted by tumor size and hormone receptors status.

	**Odds ratio (95%CI)**	**p-value**
**Baseline homocysteine (w0)**	1.135 (0.323–3.984)	0.843
Treatment arm	0.679 (0.234–1.967)	0.475
Homocysteine × treatment arm	0.976 (0.147–6.506)	0.980
		
Homocysteine w0 standard arm	0.950 (0.263–3.430)	0.937
Homocysteine w0 metformin arm	1.193 (0.265–5.373)	0.819
		
**Follow-up homocysteine (w24-w0)**	0.135 (0.009–1.983)	0.144
Treatment arm	1.400 (0.302–6.494)	0.668
Homocysteine × treatment arm	**47.584** (1.604–1411.933)	**0.026**
		
Homocysteine w24-w0 standard arm	0.144 (0.010–2.077)	0.155
Homocysteine w24-w0 metformin arm	**6.614** (0.822–53.189)	**0.076**

## DISCUSSION

We are now beginning to recognize that the causes of therapeutic cancer resistance might involve alterations in the host rather than in the cancer cells themselves. Metabolomic analysis of peripheral blood provides a snapshot of the global physiological state of several organs and tissues. We used this approach in the present study to evaluate the impact of adding metformin to a well-established neoadjuvant regimen of chemotherapy and trastuzumab on the metabolism of HER2-positive breast cancer patients. Our findings should therefore be considered in terms of the complex interaction between host and tumor, as well as on systemic effects on several metformin-responsive organs including liver, fat, and muscle.

Our results identify a signature of significantly-altered circulating metabolites that exclusively associates with the combination of metformin, chemotherapy, and trastuzumab. Moreover, we confirm that metformin can provoke a fasting-mimicking modification of the systemic host metabolism involving a significant augmentation of both the ketone body BHBA, a marker of mitochondrial fatty acid β-oxidation, and α-ketoglutarate, a key intermediate of the TCA cycle.

AMP-activated protein kinase (AMPK) and mammalian target of rapamycin (mTOR) complex 1 (mTORC1), two key regulators of metabolism that are respectively activated and inhibited in acute response to cellular energy depletion, are known to inhibit β-oxidation and ketogenesis in the liver, adipose tissue and perhaps muscle, while also promoting the use and storage of glucose [31–33]. mTORC1 blockade activates β-oxidation (i.e., adipose tissue lipolysis), thereby inducing the release of acetyl-CoA that can either enter the TCA cycle or the ketogenesis pathway when the TCA cycle is shut down (e.g., in fasting conditions) [34,35]. Our data therefore imply that one of the physiological consequences of metformin-induced inhibition of mTORC1 [36] on systemic metabolism is the release of ketone bodies, here BHBA, in the circulating metabolome of cancer patients. Circulating levels of α-ketoglutarate, which are increased by starvation and mimic calorie restriction *via* inactivation of mTOR [37], also become significantly elevated in breast cancer patients co-treated with metformin but not in those treated only with a standard combination of chemo- and targeted therapy. Upregulation of ketone body metabolism and α-ketoglutarate, both key sensors of mitochondrial perturbations that involve the mTOR pathway, provides a rationale to suggest that the partial suppression of the mitochondrial electron transport chain [38,39] by adding metformin to an established therapeutic regimen leads to a systemic catabolic response mimicking fasting in breast cancer patients. Moreover, our data suggest that HER2-positive breast cancer patients who clinically benefited from neoadjuvant metformin were particularly sensitive to its metabolic effects on mitochondrial fatty acid β-oxidation. Because breast tumor tissues were not available for metabolomic analysis we cannot discard the possibility that, beyond an indirect effect of metformin on hepatic and adipose tissues and perhaps also on short-chain fatty acid (butyrate-producing gut microbiota [40,41], it could directly promote inhibition of the mTOR pathway and increase fatty acid oxidation in the breast cancer tumor cells themselves, altogether contributing to the apparent release of BHBA into the serum of HER2-positive breast cancer patients co-treated with metformin.

Our findings also highlight the positive correlation between metformin-driven alterations in specific metabolites, here homocysteine, and the likelihood of HER2-positive breast cancer patients achieving clinical benefit from the pre-operative treatment in terms of pCR rate. We observed a significant relationship between the follow-up circulating levels of homocysteine and the ability of treatment arms to achieve pCR, suggesting that the direction and/or intensity of the relationship between the elevation of circulating homocysteine and pCR significantly varied in each treatment arm. Accordingly, those patients with significant elevations of homocysteine, a metabolic checkpoint of 1C metabolism, tended to have a significantly higher probability of pCR, but only in the metformin-containing arm. Antifolates, a group of anti-cancer agents targeting various enzymatic steps in folate-dependent 1C metabolism, are known to exert an indirect influence on the rate of appearance/disappearance of homocysteine from cellular and plasma/serum compartments [42–46]. The ability of homocysteine to behave as a shared marker of the pharmacodynamic effect of metformin and antifolate drugs strongly supports the increasing recognition that anti-diabetic biguanides may exhibit folate mimicry and antifolate-like activity [27–29,46]. Homocysteine levels are known to increase in non-cancer patients undergoing biguanide treatment [47,48], and metabolomic parallelisms have been noted between the responses of cancer cells to biguanides and anti-folate drugs such as methotrexate [27,49]. Dihydrofolate reductase, the best-understood target through which methotrexate blocks the synthesis of tetrahydrofolate methyl donors and indirectly promotes the accumulation of homocysteine [50,51], has been proposed as a putative target of metformin not only in the gut microbiota, but also in intestinal cells [28–30]. Because pre-clinical and clinical studies have shown that well-recognized detrimental effects of homocysteine such as cellular hypomethylation do not accompany the antifolate-like activity of metformin [30,52], it remains an open question whether the increase in circulating homocysteine levels, a classic marker of 1C deficiency, is secondary to reduced vitamin B12 levels, folate levels (or a combination of both), or results from direct targeting of folate-dependent enzymes in the gut microbiota, gut mucosal cells, or the tumor cells themselves. In this regard, we are currently exploring whether the ability of metformin to promote a build-up of homocysteine in those patients more likely to achieve a clinical response might be explained in terms of a non-classic disruption of 1C metabolism involving the flux of 1C units generated from serine metabolism [30,53].

Three previous clinical studies have employed metabolomic approaches to assess the pharmacodynamic effects of metformin in endometrial, ovarian, and breast cancer types. The first study involved obese, nondiabetic endometrial cancer patients (n=20) treated with metformin (850 mg) daily for up to 4 weeks prior to surgical staging in a preoperative window clinical trial for endometrial cancer. In agreement with our findings, BHBA showed the most profound change in metabolite concentration in response to metformin, and more pronounced effects were reported in the serum of responder patients [22]. The second study assayed tissue and serum samples from patients with ovarian cancer (n=10) who were receiving metformin for diabetes, while using control samples from non-diabetic patients with lower mean body-mass index [24]. The authors found that the predominant mechanism of action by metformin in cancer is to target tumor-cell intrinsic mitochondrial metabolism, as suggested by our findings of metformin-driven elevation of circulating α-ketoglutarate. The third study recruited female patients with treatment-naïve primary breast cancer (n=40) who received 13–21 days of slow release metformin at escalating dose levels (500 mg for days 1–3, 1,000 mg for days 4–6, and 1,500 mg thereafter) and lacked a control arm [25]. In agreement with our suggestion of metformin-driven β-oxidation in tumor cells, the authors found that patients with augmented glucose uptake into the primary breast cancer following metformin treatment presented a significant increase in intratumoral acetylcarnitine, likely reflecting an increased flux of glucose carbons toward acetyl-CoA *via* increased fatty acid oxidation and ketogenesis [54–56]. Nonetheless, it is important to note that our present study is the first detailing a systemic modification in host metabolism caused by metformin in cancer patients treated with targeted therapy (the anti-HER2 monoclonal antibody trastuzumab) in combination with chemotherapy (anthracycline/taxane).

In summary, recent strategies in cancer therapy have begun to focus on the potential beneficial effects of adjuvant dietary interventions (e.g., fasting, KD) on those metabolic pathways in tumor cells and the tumor environment (e.g., microbiota, tumor microenvironment, immune system) that play a key role in cancer progression and therapeutic resistance [14,57–60]. However, the safety and efficacy of such nutritional interventions should be examined for each single type/genetic subtype of cancer before they can be exploited for clinical application for cancer patients. In this context, our present findings showing that the addition of metformin to a well-established neoadjuvant regimen causes a fasting-mimicking modification of the systemic host metabolism, including an elevation of BHBA, together with the favorable safety and tolerability profile of metformin [26,61–64], might allow metformin to be considered as a moderate fasting/KD-mimicking agent in combination with standard of care therapies in multiple cancer types. Nevertheless, the ever-growing number of individual clinical trials (>300) investigating metformin in the treatment of various types of cancer has highlighted a need for more rigorous planning to focus on potential predictive biomarkers [65]. Along this line, we have recently proposed that the minor allele (C) of the single-nucleotide polymorphism (SNP) rs11212617, located near the *ataxia telangiectasia mutated* gene, might warrant consideration as a predictive clinical biomarker to inform the personalized used of metformin in breast cancer patients [66]. In contrast to predictive biomarkers, which attempt to *a priori* predict the likelihood to respond to a particular treatment from the properties of the tumor, pharmacodynamic biomarkers provide a post-treatment measure of whether a given drug has reached its target, exerted a pharmacological response, and the degree of such response [67]. In our hands, no significant relationship existed between baseline (pre-treatment) serum homocysteine levels and the ability of metformin to achieve pCR in patients, whereas the (post- minus pre-treatment) follow-up difference in circulating homocysteine across treatment paralleled the clinical efficacy of neoadjuvant metformin. Thus, circulating homocysteine might be explored as an informative, non-invasive pharmacodynamic biomarker capable of linking the antifolate-like activity of metformin and biological tumor response using other treatment regimens and other cancer types.

## MATERIALS AND METHODS

### Participants

We prospectively collected serum samples from patients (*n*=68) with early, non-metastatic HER2-positive breast cancer that were recruited into the METTEN study (EU Clinical Trials Register, EudraCT number 2011-000490-30; registered 28 February 2011, https://www.clinicaltrialsregister.eu/ctr-search/trial/2011-000490-30/ES) [26]. The ethics committee of the Dr. Josep Trueta Hospital (Girona, Spain) and independent Institutional Review Boards at each site participating in the METTEN study approved the protocol and any amendments. All procedures were in accordance with the ethical standards of the institutional research committees and with the 1964 Helsinki Declaration and its later amendments or comparable ethical standards. Informed consent was obtained from all individual participants included in the metabolomic sub-study presented here.

Patients were randomly assigned to receive daily metformin (850 mg twice-daily) for 24 weeks concurrently with 12 cycles of weekly paclitaxel (80 mg/m^2^) plus trastuzumab (4 mg/kg loading dose followed by 2 mg/kg) followed by four cycles of 3-weekly fluorouracil (600 mg/m^2^), epirubicin (75 mg/m^2^), cyclophosphamide (600 mg/m^2^) with concomitant trastuzumab (6 mg/kg) (arm A), or equivalent sequential chemotherapy plus trastuzumab without metformin (arm B), followed by surgery. Patients had surgery within 4–5 weeks of the last cycle of neoadjuvant treatment [26]. In all participants, venous blood was collected, after an overnight fast, into tubes with no added anticoagulants (serum). The tubes were centrifuged at 2500 × g at 4^o^C, and serum was stored at -80^o^C until use to minimize preanalytical errors. Post-surgery, patients received thrice-weekly trastuzumab to complete 1 year of neoadjuvant-adjuvant therapy.

### Metabolomics

Methods to optimize reproducibility and robustness for the simultaneous measurement of selected metabolites from energy and 1C metabolism and chromatographic conditions have been previously described [68–70]. Briefly, surrogate deuterated standards were added to maximize technical precision during the injection and recovery during the extraction procedures (Isotec Stable Isotopes, Miamisburg, OH, USA). The calibration curves were prepared immediately before each assay using commercially available metabolites (Fluka, St Gallen, Switzerland). The samples for gas chromatography were derivatized and analyzed on an Agilent Technologies (Santa Clara, CA, USA) 7890A gas chromatograph coupled with an electron impact (EI) source to a 7200 quadrupole time-of-flight mass spectrometer (QTOF-MS) equipped with a 7693 auto-sampler module and a J&W Scientific HP-5MS column (30 m × 0.25 mm, 0.25 μm). The liquid chromatography platform (UHPLC-ESI-QqQ-MS) was based on an Agilent 1290 Infinity Ultra High Performance Liquid Chromatograph (UHPLC) coupled with an iFunnel electrospray ionization source (ESI) and a 6490 triple quadrupole mass spectrometer (QqQ-MS). The MS analysis alternated between MS and data-dependent MS^2^ scans using dynamic exclusion. Metabolites were identified and quantified using available reference libraries and the Qualitative and Quantitative Analysis B.06.00 software (Agilent Technologies).

### Statistical analysis

Descriptive data were summarized using percentages, medians or means with their respective 25 and 75 percentiles, or standard deviations as appropriate. Clinical baseline characteristics between treatment arms were assessed using Chi-square or Fisher’s exact test for categorical variables, Student’s *t* test for continuous variables with normal distribution, or Mann-Whitney *U* test for non-normal distributions. The assumption of normality was evaluated with the Shapiro-Wilk test. Changes in circulating metabolite levels between pre- and post-treatment were compared using the Wilcoxon test. Binary logistic regression was used to assess the prognostic effect of both the baseline and the follow-up (post-pre) differences in circulating metabolites on pCR. Unadjusted and adjusted ORs with their relative 95% CIs were reported as a measure of association. All tests were 2-sided and p≤0.05 was set as statistically significant. Statistical analyses were carried out using SPSS (IBM Corp. released 2017. IBM SPSS Statistics for Windows, Version 25.0; Armonk, NY, USA) and STATA (StataCorp. 2013. Stata Statistical Software: Release 13; StataCorp LP, College Station, TX, USA).
